# Aqua­bis­(1,1,1,5,5,5-hexa­fluoro­acetyl­acetonato)[4′-(4-pyrid­yl)-2,2′:6′,2′′-terpyridine]­ytterbium(III) chloride methanol monosolvate monohydrate

**DOI:** 10.1107/S1600536811052378

**Published:** 2011-12-10

**Authors:** Toru Okawara, Jiang Feng, Masaaki Abe, Yoshio Hisaeda

**Affiliations:** a Moto-oka 744, Nishi-ku, Fukuoka 819-0395, Japan

## Abstract

The title compound, [Yb(C_5_HF_6_O_2_)_2_(C_20_H_14_N_4_)(H_2_O)]Cl·CH_3_OH·H_2_O, adopts an eight-coordinated geometry around the Yb^III^ atom consisting of a 4′-(4-pyrid­yl)-2,2′:6′,2′′-terpyridine (pytpy) ligand, two 1,1,1,5,5,5-hexa­fluoro­acetyl­acetonate (hfac) anions and an aqua ligand. In the solid state, the compound forms supra­molecular chains running along the *b*-axis *via* inter­molecular hydrogen bonds between the Yb—OH_2_ unit and the N-atom donor of the 4-pyridyl pendant of pytpy, with an O⋯N distance of 2.686 (4) Å. A chloride counter-anion and lattice methanol and water solvent mol­ecules occupy a hydro­philic columnar space along the coordination chains. O—H⋯Cl hydrogen bonds occur. The two water molecules and the four trifluoromethyl groups are disordered over two sets of sites, each with different occupancy ratios.

## Related literature

For general background to pytpy, see: Constable & Thompson (1992 ▶, 1994 ▶). For pytpy complexes, see: Sun *et al.* (2000 ▶); Sun & Lees (2001 ▶). For related Yb complexes, see: Fukuda *et al.* (2002 ▶); Hayashi *et al.* (1998 ▶); Przychodzen *et al.* (2007 ▶); Stojanovic *et al.* (2010 ▶); Li *et al.* (2007 ▶); Xu *et al.* (2009 ▶); Ahrens *et al.* (2002 ▶); Zhang *et al.* (2007*a* ▶). For potential applications of compounds with infinite one-dimensional to three-dimensional structures, see: Hayami *et al.* (2004 ▶); Hou *et al.* (2005 ▶); Feng *et al.* (2006 ▶); Beves *et al.* (2007*a* ▶); Zhang *et al.* (2007*b* ▶); Gou *et al.* (2008 ▶); Leong & Vittal (2011 ▶); Moulton & Zaworotko (2001 ▶). For the binding mode of pytpy involving hydrogen-bonding, see: Beves *et al.* (2007*b* ▶, 2008 ▶).
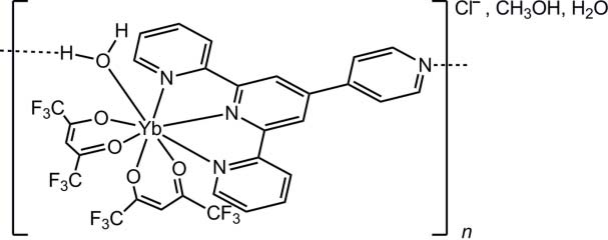


## Experimental

### 

#### Crystal data

[Yb(C_5_HF_6_O_2_)_2_(C_20_H_14_N_4_)(H_2_O)]Cl·CH_4_O·H_2_O
*M* *_r_* = 1001.03
Triclinic, 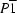

*a* = 9.7559 (6) Å
*b* = 12.4035 (7) Å
*c* = 16.5543 (10) Å
α = 98.870 (1)°
β = 104.717 (1)°
γ = 93.559 (1)°
*V* = 1903.5 (2) Å^3^
*Z* = 2
Mo *K*α radiation
μ = 2.63 mm^−1^
*T* = 223 K
0.46 × 0.33 × 0.16 mm

#### Data collection

Bruker SMART APEX CCD diffractometer
Absorption correction: multi-scan (*SADABS*; Bruker, 2008 ▶) *T*_min_ = 0.53, *T*_max_ = 0.68
13523 measured reflections
9671 independent reflections
8205 reflections with *I* > 2σ(*I*)
*R*_int_ = 0.021

#### Refinement

*R*[*F* ^2^ > 2σ(*F* ^2^)] = 0.036
*wR*(*F* ^2^) = 0.081
*S* = 1.08
9671 reflections
587 parameters
34 restraints
H atoms treated by a mixture of independent and constrained refinement
Δρ_max_ = 1.02 e Å^−3^
Δρ_min_ = −0.88 e Å^−3^

### 

Data collection: *APEX2* (Bruker, 2010 ▶); cell refinement: *SAINT* (Bruker, 2009 ▶); data reduction: *SAINT*; program(s) used to solve structure: *SHELXS97* (Sheldrick, 2008 ▶); program(s) used to refine structure: *SHELXL97* (Sheldrick, 2008 ▶); molecular graphics: *CrystalMaker* (*CrystalMaker*, 2010 ▶); software used to prepare material for publication: *publCIF* (Westrip, 2010 ▶).

## Supplementary Material

Crystal structure: contains datablock(s) global, I. DOI: 10.1107/S1600536811052378/mw2026sup1.cif

Structure factors: contains datablock(s) I. DOI: 10.1107/S1600536811052378/mw2026Isup2.hkl

Additional supplementary materials:  crystallographic information; 3D view; checkCIF report

## Figures and Tables

**Table 1:** Hydrogen-bond geometry (Å, °)

*D*—H⋯*A*	*D*—H	H⋯*A*	*D*⋯*A*	*D*—H⋯*A*
O5—H5*B*⋯Cl1^i^	0.75 (4)	2.31 (4)	3.054 (3)	175 (5)
O6—H6⋯Cl1	0.83	2.27	3.102 (3)	177
O5—H5*A*⋯N4^ii^	0.78 (4)	1.92 (4)	2.686 (4)	167 (5)

Symmetry codes: (i) 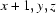; (ii) 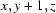.

**Fig. 1.**
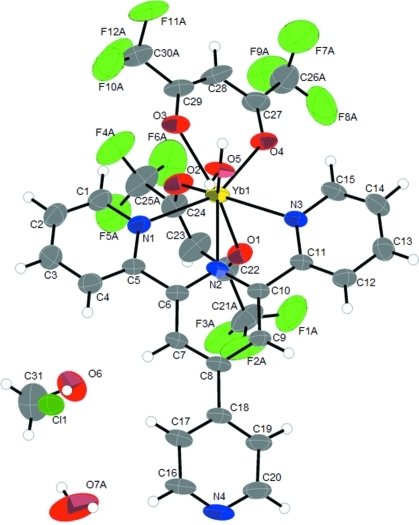
An ORTEP view of the title compound, with the atom-labelling scheme. Displacement ellipsoids are drawn at the 50% probability level. The minor component of the disordered CF3 groups and lattice water are omitted for clarity.

## supplementary crystallographic information

### Comment

The molecular design of multidentate ligands is crucial to determining structures and functions of the resulting coordination compounds and metallo-supramolecular systems. Specifically, a tetradentate ligand 4'-(4-pyridyl)-2,2':6',2"-terpyridine (pytpy) provides a unique structural feature as a bridging ligand where two different coordination donors, the tridentate terpyridyl and monodentate pyridyl moieties, are both associated with metal coordination. Herein we report an unusual bridging mode of pytpy in a one-dimensional metallo-supramolecular system as exemplified with an X-ray crystal structure of compound (I), where the monodentate pyridyl arm in pytpy is now bound to the neighboring molecule *via* intermolecular hydrogen bonding to form a one-dimensional supramolecular chain. Compound (I) consists of a monocationic complex [Yb^III^(pytpy)(hfac)_2_(H_2_O)], a Cl^-^ anion, and lattice solvents, CH_3_OH and H_2_O. The Yb^III^ center is surrounded by three N donors from pytpy and five O donors from two hfac chelates and one aqua ligand completing the 8-coordinate geometry as shown in Figure 1. Among structurally determined Yb^III^ complexes containing a single terpyridine ligand, the coordination number 8 is rather unusual and 9- and 10-coordination is more commonly observed (Hayashi *et al.*, 1998; Ahrens *et al.*, 2002; Fukuda *et al.*, 2002; Przychodzen *et al.*, 2007; Li *et al.*, 2007; Xu *et al.*, 2009; Stojanovic *et al.*, 2010). The 8-coordination around lanthanide(III) ions are seen, for example, in [Ln^III^(Trop)_4_]^-^ [Trop = tropolonene (2-hydroxycyclohepta-2,4,6-trienone)] (Zhang *et al.*, 2007*a*). In compound (I), the Yb—N(pytpy) lengths vary from 2.434 (3) to 2.464 (3) Å and the Yb—O(hfac) lengths from 2.262 (3) to 2.334 (3) Å; these values compare well with those observed in complexes containing the [Yb^III^(tpy)(hfac)_3_] entity (Li *et al.*, 2007; Xu *et al.*, 2009). There is a hydrogen-bonding interaction with the chloride anion with an O5···Cl1^i^ (symmetry code: (i) 1 + *x*, *y*, *z*) distance of 3.054 (3) Å and an O6···Cl1 distance of 3.102 (3) Å. An additional hydrogen-bonding interaction is seen between the N atom of the dangling pyridyl group and the aqua ligand in the neighboring molecule with an O5···N4^ii^ (symmetry code: (ii) *x*, 1 + *y*, *z*) distance of 2.686 (4) Å to form one-dimensional supramolecular chains of [Yb(pytpy)(hfac)_2_(H_2_O)]^+^ units running along the *b*-axis. Similar hydrogen bonded one-dimensional networks including pytpy moieties have been also reported (Beves *et al.*, 2007*b*; Beves *et al.*, 2008).

### Experimental

An ethanol solution (50 ml) of pytpy (600 mg, 1.94 mmol) Hhfac (1.21 g, 5.92 mmol), and YbCl_3_^.^6H_2_O (760 mg, 1.94 mmol) was stirred for 30 min at room temperature. After evaporation, the residue was recrystallized from CH_3_OH/water to give (I) as colorless crystals. Elemental analysis of the compound that was dried by vacuum pumping overnight at room temperature reveals the loss of the solvent molecules of crystallization (H_2_O and CH_3_OH). Transparent needle-shaped single crystals of compound (I) suitable for X-ray diffraction analysis were obtained by slow evaporation of a CH_3_OH/water (95:5, *v*/*v*) solution in a few days. Yield: 637 mg, 0.67 mmol (35%). Analysis: calculated for C_30_H_18_ClF_12_N_4_O_5_Yb ([Yb(pytpy)(hfac)_2_(H_2_O)]Cl): C 37.89, H 1.91, N 5.89%; found: C 37.60, H 1.96, N 5.92%. IR (KBr pellet): 1603, 1664, 3031, 3410 cm^-1^. UV-vis (CH_3_OH) *λ*max/nm (*ε*/*M*^-1^cm^-1^): 241 (41,300), 285 (34,300). ESI-TOF-MS (CH_3_OH): *m*/*z* 898.39 (calcd: 898.04 for [M–2H_2_O–CH_3_OH–Cl^-^]^+^).

### Refinement

H atoms except those of water were placed in geometrically idealized positions and constrained to ride on their parent atoms with *U*_iso_(H) = 1.2*U*_eq_(C—H) or 1.5*U*_eq_(O—H).

The lattice water shows positional disorder which is modeled as two oxygen atoms, O7A and O7B, with site occupancies of 0.58 and 0.42, respectively. The O7A—O7A^iii^ (symmetry codes: (iii) 1 - *x*, 2 - *y*, 1 - *z*) distance was restrained to 2.56 (1) Å using the *DFIX* command of the program *SHELXTL* (Sheldrick, 2008) because of a strong correlation between positional parameters of the two components of the disorder.

H atoms attached to O5 (H5A and H5B) and lattice water (H7A, H7B, H7C, and H7D) were found in a difference Fourier map. The O—H and H—H distances within the water molecules were restrained to 0.83 (7) Å) and 1.35 (8) Å , respectively, by using the *DFIX* command for a stable refinement. Hydrogen atoms on the lattice water were not included in the structure factor calculation.

Four trifluoromethyl groups were found to show disorder. The geometries of the trifluoromethyl groups were constrained by using the SAME command. Anisotropic displacement parameters of the pairs of overlapping disordered atoms of the major and minor components of the disorder were made equal using the EADP constraints. The final occupancies of the disordered CF_3_ groups were found to be 0.81:0.19, 0.76:0.24, 0.90:0.10, and 0.86:0.14 for (C21A, F1A, F2A, F3A)/(C21B, F1B, F2B, F3B), (C25A, F4A, F5A, F6A)/(C25B, F4B, F5B, F6B), (C26A, F7A, F8A, F9A)/(C26B, F7B, F8B, F9B), and (C30A, F10A, F11A, F12A)/(C30B, F10B, F11B, F12B), respectively.

### Crystal data

**Table:**

[Yb(C_5_HF_6_O_2_)_2_(C_20_H_14_N_4_)(H_2_O)]Cl·CH_4_O·H_2_O	*Z* = 2
*M**_r_* = 1001.03	*F*(000) = 978
Triclinic, *P*1	*D*_x_ = 1.747 Mg m^−^^3^
*a* = 9.7559 (6) Å	Mo *K*α radiation, λ = 0.71073 Å
*b* = 12.4035 (7) Å	Cell parameters from 4823 reflections
*c* = 16.5543 (10) Å	θ = 2.6–28.9°
α = 98.870 (1)°	µ = 2.63 mm^−^^1^
β = 104.717 (1)°	*T* = 223 K
γ = 93.559 (1)°	Prism, colourless
*V* = 1903.5 (2) Å^3^	0.46 × 0.33 × 0.16 mm

### Data collection

**Table:**

Bruker SMART APEX CCD diffractometer	9671 independent reflections
Radiation source: fine focus sealed tube	8205 reflections with *I* > 2σ(*I*)
graphite	*R*_int_ = 0.021
Detector resolution: 8.3333 pixels mm^-1^	θ_max_ = 28.7°, θ_min_ = 1.7°
phi and ω scans	*h* = −13→13
Absorption correction: multi-scan (*SADABS*; Bruker, 2008)	*k* = −13→16
*T*_min_ = 0.53, *T*_max_ = 0.68	*l* = −22→20
13523 measured reflections	

### Refinement

**Table:**

Refinement on *F*^2^	Primary atom site location: structure-invariant direct methods
Least-squares matrix: full	Secondary atom site location: difference Fourier map
*R*[*F*^2^ > 2σ(*F*^2^)] = 0.036	Hydrogen site location: inferred from neighbouring sites
*wR*(*F*^2^) = 0.081	H atoms treated by a mixture of independent and constrained refinement
*S* = 1.08	*w* = 1/[σ^2^(*F*_o_^2^) + (0.030*P*)^2^ + 1.1*P*] where *P* = (*F*_o_^2^ + 2*F*_c_^2^)/3
9671 reflections	(Δ/σ)_max_ = 0.001
587 parameters	Δρ_max_ = 1.02 e Å^−^^3^
34 restraints	Δρ_min_ = −0.88 e Å^−^^3^

### Special details

**Table:**

Geometry. All e.s.d.'s (except the e.s.d. in the dihedral angle between two l.s. planes) are estimated using the full covariance matrix. The cell e.s.d.'s are taken into account individually in the estimation of e.s.d.'s in distances, angles and torsion angles; correlations between e.s.d.'s in cell parameters are only used when they are defined by crystal symmetry. An approximate (isotropic) treatment of cell e.s.d.'s is used for estimating e.s.d.'s involving l.s. planes.
Refinement. Refinement of *F*^2^ against ALL reflections. The weighted *R*-factor *wR* and goodness of fit *S* are based on *F*^2^, conventional *R*-factors *R* are based on *F*, with *F* set to zero for negative *F*^2^. The threshold expression of *F*^2^ > σ(*F*^2^) is used only for calculating *R*-factors(gt) *etc*. and is not relevant to the choice of reflections for refinement. *R*-factors based on *F*^2^ are statistically about twice as large as those based on *F*, and *R*- factors based on ALL data will be even larger.

### Fractional atomic coordinates and isotropic or equivalent isotropic displacement parameters (Å^2^)

**Table:**

	*x*	*y*	*z*	*U*_iso_*/*U*_eq_	Occ. (<1)
O7A	0.4270 (10)	0.9463 (10)	0.5369 (8)	0.135 (6)	0.584 (19)
H7A	0.4377	0.9366	0.489	0.203*	0.58
H7B	0.4045	0.9042	0.5548	0.203*	0.58
O7B	0.4211 (17)	0.9784 (8)	0.5886 (11)	0.101 (6)	0.416 (19)
H7C	0.4744	0.9843	0.633	0.151*	0.42
H7D	0.3432	0.9793	0.5672	0.151*	0.42
F1A	0.5633 (6)	0.4015 (4)	0.0987 (6)	0.132 (3)	0.809 (7)
F2A	0.7562 (8)	0.3375 (4)	0.1479 (3)	0.115 (2)	0.809 (7)
F3A	0.6812 (8)	0.3306 (5)	0.0145 (3)	0.106 (2)	0.809 (7)
C21A	0.6903 (11)	0.3909 (7)	0.0894 (4)	0.078 (2)	0.809 (7)
F4A	0.9969 (9)	0.7609 (6)	−0.0307 (5)	0.095 (2)	0.760 (10)
F5A	0.9299 (10)	0.5959 (5)	−0.0973 (3)	0.104 (2)	0.760 (10)
F6A	0.7780 (7)	0.7104 (9)	−0.0868 (5)	0.127 (3)	0.760 (10)
C25A	0.8965 (10)	0.6797 (7)	−0.0462 (5)	0.072 (2)	0.760 (10)
F7A	0.6123 (6)	1.0312 (4)	0.1179 (4)	0.136 (3)	0.901 (7)
F8A	0.5579 (6)	0.9439 (5)	0.2082 (4)	0.133 (2)	0.901 (7)
F9A	0.5449 (5)	0.8597 (4)	0.0849 (4)	0.126 (2)	0.901 (7)
C26A	0.6202 (8)	0.9367 (7)	0.1460 (6)	0.088 (2)	0.901 (7)
F10A	1.2675 (6)	1.0106 (4)	0.2103 (4)	0.1137 (19)	0.861 (6)
F11A	1.1197 (5)	1.1250 (3)	0.1800 (3)	0.0930 (14)	0.861 (6)
F12A	1.1272 (7)	0.9944 (5)	0.0843 (3)	0.136 (3)	0.861 (6)
C30A	1.1366 (8)	1.0194 (5)	0.1657 (5)	0.0693 (18)	0.861 (6)
F1B	0.547 (2)	0.4106 (19)	0.0297 (19)	0.132 (3)	0.191 (7)
F2B	0.654 (4)	0.3824 (19)	0.1497 (14)	0.115 (2)	0.191 (7)
F3B	0.724 (3)	0.328 (3)	0.0454 (16)	0.106 (2)	0.191 (7)
C21B	0.672 (3)	0.413 (3)	0.0808 (18)	0.078 (2)	0.191 (7)
F4B	1.023 (3)	0.727 (2)	−0.0193 (17)	0.095 (2)	0.240 (10)
F5B	0.844 (3)	0.627 (2)	−0.1050 (12)	0.104 (2)	0.240 (10)
F6B	0.808 (2)	0.774 (2)	−0.0447 (14)	0.127 (3)	0.240 (10)
C25B	0.889 (3)	0.697 (2)	−0.0299 (18)	0.072 (2)	0.240 (10)
F7B	0.643 (6)	1.055 (4)	0.168 (3)	0.136 (3)	0.099 (7)
F8B	0.548 (6)	0.892 (4)	0.153 (4)	0.133 (2)	0.099 (7)
F9B	0.615 (4)	0.944 (4)	0.054 (3)	0.126 (2)	0.099 (7)
C26B	0.650 (6)	0.951 (4)	0.137 (3)	0.088 (2)	0.099 (7)
F10B	1.237 (4)	1.053 (3)	0.215 (2)	0.1137 (19)	0.139 (6)
F11B	1.095 (3)	1.080 (2)	0.109 (2)	0.0930 (14)	0.139 (6)
F12B	1.203 (4)	0.931 (3)	0.102 (2)	0.136 (3)	0.139 (6)
C30B	1.142 (5)	0.998 (3)	0.147 (2)	0.0693 (18)	0.139 (6)
C1	1.2599 (5)	0.6952 (3)	0.1920 (3)	0.0523 (10)	
H1	1.2293	0.7561	0.1676	0.063*	
C2	1.3902 (5)	0.6611 (3)	0.1860 (3)	0.0537 (10)	
H2	1.4462	0.6976	0.1579	0.064*	
C3	1.4353 (5)	0.5733 (4)	0.2220 (3)	0.0537 (10)	
H3	1.5237	0.5489	0.2195	0.064*	
C4	1.3497 (4)	0.5204 (3)	0.2621 (3)	0.0468 (9)	
H4	1.3795	0.4598	0.2872	0.056*	
C5	1.2205 (4)	0.5574 (3)	0.2650 (2)	0.0361 (8)	
C6	1.1229 (4)	0.5037 (3)	0.3055 (2)	0.0342 (7)	
C7	1.1385 (4)	0.3992 (3)	0.3247 (2)	0.0388 (8)	
H7	1.213	0.3609	0.3123	0.047*	
C8	1.0437 (4)	0.3513 (3)	0.3624 (2)	0.0383 (8)	
C9	0.9384 (4)	0.4127 (3)	0.3816 (2)	0.0369 (8)	
H9	0.8752	0.3845	0.4095	0.044*	
C10	0.9266 (4)	0.5166 (3)	0.3593 (2)	0.0338 (7)	
C11	0.8142 (4)	0.5847 (3)	0.3771 (2)	0.0347 (7)	
C12	0.7307 (4)	0.5584 (3)	0.4284 (2)	0.0417 (8)	
H12	0.7421	0.4944	0.4522	0.05*	
C13	0.6296 (5)	0.6267 (3)	0.4449 (3)	0.0501 (10)	
H13	0.5704	0.6092	0.4788	0.06*	
C14	0.6188 (5)	0.7210 (3)	0.4099 (3)	0.0519 (10)	
H14	0.5534	0.77	0.4208	0.062*	
C15	0.7043 (5)	0.7420 (3)	0.3593 (3)	0.0474 (10)	
H15	0.6955	0.8066	0.336	0.057*	
C16	1.1819 (5)	0.0805 (3)	0.3982 (3)	0.0571 (12)	
H16	1.2672	0.0481	0.401	0.068*	
C17	1.1783 (5)	0.1875 (3)	0.3838 (3)	0.0505 (10)	
H17	1.2592	0.2262	0.3768	0.061*	
C18	1.0535 (4)	0.2369 (3)	0.3799 (2)	0.0399 (8)	
C19	0.9383 (5)	0.1753 (3)	0.3908 (3)	0.0519 (10)	
H19	0.8517	0.2055	0.3887	0.062*	
C20	0.9520 (6)	0.0688 (3)	0.4049 (3)	0.0594 (12)	
H20	0.8731	0.0281	0.4126	0.071*	
C22	0.7704 (5)	0.5083 (3)	0.0997 (3)	0.0523 (10)	
C23	0.8004 (6)	0.5427 (4)	0.0297 (3)	0.0718 (15)	
H23	0.7685	0.4974	−0.0239	0.086*	
C24	0.8767 (5)	0.6429 (4)	0.0383 (3)	0.0515 (10)	
C27	0.7744 (5)	0.9089 (3)	0.1768 (3)	0.0569 (11)	
C28	0.8842 (5)	0.9693 (4)	0.1609 (3)	0.0655 (13)	
H28	0.8637	1.0278	0.1314	0.079*	
C29	1.0244 (5)	0.9468 (3)	0.1870 (3)	0.0534 (11)	
C31	0.4832 (7)	0.7478 (6)	0.7308 (4)	0.0958 (19)	
H31A	0.5547	0.7518	0.7843	0.144*	
H31B	0.397	0.7054	0.7319	0.144*	
H31C	0.4623	0.8213	0.7223	0.144*	
Cl1	0.30493 (11)	0.69937 (8)	0.49683 (7)	0.0517 (2)	
N1	1.1748 (3)	0.6454 (2)	0.23092 (19)	0.0405 (7)	
N2	1.0163 (3)	0.5603 (2)	0.32052 (17)	0.0329 (6)	
N3	0.8006 (3)	0.6758 (2)	0.34053 (18)	0.0376 (7)	
N4	1.0714 (5)	0.0212 (3)	0.4082 (2)	0.0552 (9)	
O1	0.8032 (3)	0.5596 (2)	0.17364 (16)	0.0442 (6)	
O2	0.9332 (3)	0.7075 (2)	0.10503 (16)	0.0512 (7)	
O3	1.0696 (3)	0.8734 (2)	0.22719 (16)	0.0476 (7)	
O4	0.7822 (3)	0.8267 (2)	0.21397 (18)	0.0505 (7)	
O5	1.0781 (3)	0.8027 (2)	0.37801 (17)	0.0458 (7)	
H5A	1.087 (5)	0.867 (3)	0.392 (3)	0.069*	
H5B	1.134 (5)	0.775 (4)	0.405 (3)	0.069*	
O6	0.5339 (4)	0.6980 (3)	0.6653 (2)	0.0727 (10)	
H6	0.4737	0.6963	0.6195	0.109*	
Yb1	0.953387 (18)	0.714155 (11)	0.248364 (9)	0.03543 (6)	

### Atomic displacement parameters (Å^2^)

**Table:**

	*U*^11^	*U*^22^	*U*^33^	*U*^12^	*U*^13^	*U*^23^
O7A	0.129 (9)	0.068 (7)	0.177 (13)	−0.022 (6)	−0.026 (8)	0.055 (8)
O7B	0.163 (13)	0.047 (6)	0.130 (12)	0.040 (7)	0.101 (11)	0.015 (6)
F1A	0.100 (4)	0.105 (4)	0.186 (8)	−0.051 (3)	0.076 (5)	−0.023 (5)
F2A	0.190 (7)	0.054 (3)	0.094 (3)	−0.032 (3)	0.029 (4)	0.021 (2)
F3A	0.169 (6)	0.074 (2)	0.057 (3)	−0.061 (3)	0.035 (3)	−0.024 (3)
C21A	0.109 (6)	0.064 (5)	0.052 (4)	−0.040 (4)	0.028 (3)	−0.009 (3)
F4A	0.121 (4)	0.105 (5)	0.055 (3)	−0.038 (4)	0.016 (3)	0.036 (3)
F5A	0.146 (7)	0.118 (4)	0.054 (2)	−0.003 (4)	0.048 (4)	0.003 (2)
F6A	0.104 (4)	0.196 (9)	0.085 (5)	−0.001 (5)	−0.009 (4)	0.098 (5)
C25A	0.099 (5)	0.081 (5)	0.025 (4)	−0.027 (4)	0.008 (3)	0.004 (3)
F7A	0.094 (4)	0.102 (4)	0.228 (8)	0.039 (3)	0.012 (4)	0.117 (5)
F8A	0.110 (4)	0.158 (5)	0.180 (6)	0.089 (3)	0.073 (4)	0.092 (4)
F9A	0.080 (3)	0.119 (4)	0.152 (5)	0.005 (3)	−0.029 (3)	0.044 (3)
C26A	0.072 (5)	0.078 (4)	0.133 (6)	0.026 (4)	0.025 (4)	0.075 (5)
F10A	0.069 (3)	0.105 (4)	0.187 (5)	0.001 (3)	0.032 (3)	0.092 (4)
F11A	0.126 (3)	0.047 (2)	0.105 (3)	−0.020 (2)	0.024 (3)	0.032 (2)
F12A	0.196 (7)	0.123 (6)	0.095 (4)	−0.066 (4)	0.086 (4)	−0.010 (3)
C30A	0.078 (4)	0.058 (4)	0.073 (5)	−0.010 (3)	0.015 (3)	0.031 (3)
F1B	0.100 (4)	0.105 (4)	0.186 (8)	−0.051 (3)	0.076 (5)	−0.023 (5)
F2B	0.190 (7)	0.054 (3)	0.094 (3)	−0.032 (3)	0.029 (4)	0.021 (2)
F3B	0.169 (6)	0.074 (2)	0.057 (3)	−0.061 (3)	0.035 (3)	−0.024 (3)
C21B	0.109 (6)	0.064 (5)	0.052 (4)	−0.040 (4)	0.028 (3)	−0.009 (3)
F4B	0.121 (4)	0.105 (5)	0.055 (3)	−0.038 (4)	0.016 (3)	0.036 (3)
F5B	0.146 (7)	0.118 (4)	0.054 (2)	−0.003 (4)	0.048 (4)	0.003 (2)
F6B	0.104 (4)	0.196 (9)	0.085 (5)	−0.001 (5)	−0.009 (4)	0.098 (5)
C25B	0.099 (5)	0.081 (5)	0.025 (4)	−0.027 (4)	0.008 (3)	0.004 (3)
F7B	0.094 (4)	0.102 (4)	0.228 (8)	0.039 (3)	0.012 (4)	0.117 (5)
F8B	0.110 (4)	0.158 (5)	0.180 (6)	0.089 (3)	0.073 (4)	0.092 (4)
F9B	0.080 (3)	0.119 (4)	0.152 (5)	0.005 (3)	−0.029 (3)	0.044 (3)
C26B	0.072 (5)	0.078 (4)	0.133 (6)	0.026 (4)	0.025 (4)	0.075 (5)
F10B	0.069 (3)	0.105 (4)	0.187 (5)	0.001 (3)	0.032 (3)	0.092 (4)
F11B	0.126 (3)	0.047 (2)	0.105 (3)	−0.020 (2)	0.024 (3)	0.032 (2)
F12B	0.196 (7)	0.123 (6)	0.095 (4)	−0.066 (4)	0.086 (4)	−0.010 (3)
C30B	0.078 (4)	0.058 (4)	0.073 (5)	−0.010 (3)	0.015 (3)	0.031 (3)
C1	0.066 (3)	0.045 (2)	0.054 (3)	0.006 (2)	0.025 (2)	0.0186 (19)
C2	0.062 (3)	0.052 (2)	0.052 (3)	−0.002 (2)	0.024 (2)	0.0137 (19)
C3	0.047 (2)	0.053 (2)	0.060 (3)	0.0008 (19)	0.015 (2)	0.008 (2)
C4	0.045 (2)	0.038 (2)	0.055 (2)	0.0025 (17)	0.0080 (18)	0.0101 (17)
C5	0.045 (2)	0.0238 (16)	0.0355 (18)	0.0010 (14)	0.0053 (15)	0.0042 (13)
C6	0.043 (2)	0.0243 (15)	0.0320 (17)	0.0026 (14)	0.0032 (14)	0.0056 (13)
C7	0.048 (2)	0.0264 (16)	0.042 (2)	0.0099 (15)	0.0103 (16)	0.0068 (14)
C8	0.054 (2)	0.0234 (16)	0.0355 (18)	0.0083 (15)	0.0053 (16)	0.0079 (13)
C9	0.050 (2)	0.0264 (16)	0.0368 (19)	0.0054 (15)	0.0126 (16)	0.0111 (13)
C10	0.046 (2)	0.0237 (15)	0.0304 (17)	0.0030 (14)	0.0075 (15)	0.0067 (12)
C11	0.046 (2)	0.0241 (15)	0.0325 (17)	0.0049 (14)	0.0059 (15)	0.0069 (13)
C12	0.057 (2)	0.0332 (18)	0.0369 (19)	0.0049 (16)	0.0122 (17)	0.0110 (15)
C13	0.057 (3)	0.049 (2)	0.051 (2)	0.0112 (19)	0.023 (2)	0.0108 (18)
C14	0.060 (3)	0.047 (2)	0.054 (2)	0.023 (2)	0.019 (2)	0.0112 (19)
C15	0.063 (3)	0.0336 (19)	0.049 (2)	0.0200 (18)	0.014 (2)	0.0127 (16)
C16	0.071 (3)	0.0290 (19)	0.067 (3)	0.018 (2)	0.005 (2)	0.0147 (18)
C17	0.062 (3)	0.0288 (18)	0.058 (3)	0.0116 (18)	0.008 (2)	0.0122 (17)
C18	0.061 (2)	0.0241 (16)	0.0354 (19)	0.0094 (16)	0.0108 (17)	0.0090 (13)
C19	0.072 (3)	0.0307 (19)	0.062 (3)	0.0158 (19)	0.026 (2)	0.0194 (18)
C20	0.088 (4)	0.031 (2)	0.069 (3)	0.012 (2)	0.031 (3)	0.0190 (19)
C22	0.056 (3)	0.050 (2)	0.044 (2)	−0.009 (2)	0.0093 (19)	0.0007 (18)
C23	0.095 (4)	0.071 (3)	0.037 (2)	−0.030 (3)	0.013 (2)	−0.005 (2)
C24	0.059 (3)	0.056 (3)	0.037 (2)	−0.001 (2)	0.0057 (18)	0.0138 (18)
C27	0.067 (3)	0.043 (2)	0.067 (3)	0.017 (2)	0.013 (2)	0.029 (2)
C28	0.075 (3)	0.044 (2)	0.081 (3)	0.008 (2)	0.010 (3)	0.041 (2)
C29	0.072 (3)	0.034 (2)	0.051 (2)	−0.0040 (19)	0.006 (2)	0.0188 (17)
C31	0.079 (4)	0.129 (5)	0.077 (4)	0.018 (4)	0.014 (3)	0.024 (4)
Cl1	0.0523 (6)	0.0507 (6)	0.0543 (6)	0.0140 (5)	0.0087 (5)	0.0223 (4)
N1	0.053 (2)	0.0318 (15)	0.0399 (17)	0.0038 (14)	0.0136 (14)	0.0126 (12)
N2	0.0403 (16)	0.0218 (13)	0.0337 (15)	0.0062 (11)	0.0027 (12)	0.0068 (11)
N3	0.0490 (18)	0.0259 (14)	0.0387 (16)	0.0081 (13)	0.0097 (14)	0.0095 (12)
N4	0.090 (3)	0.0249 (15)	0.050 (2)	0.0142 (17)	0.0127 (19)	0.0116 (14)
O1	0.0527 (17)	0.0392 (14)	0.0368 (14)	−0.0025 (12)	0.0065 (12)	0.0065 (11)
O2	0.071 (2)	0.0422 (15)	0.0349 (14)	−0.0060 (13)	0.0037 (13)	0.0133 (11)
O3	0.0636 (18)	0.0328 (13)	0.0442 (15)	0.0035 (12)	0.0046 (13)	0.0174 (11)
O4	0.0563 (18)	0.0412 (15)	0.0595 (18)	0.0145 (13)	0.0115 (14)	0.0283 (13)
O5	0.0644 (19)	0.0223 (12)	0.0413 (15)	0.0080 (12)	−0.0051 (13)	0.0077 (10)
O6	0.059 (2)	0.087 (2)	0.070 (2)	0.0292 (19)	0.0039 (17)	0.0225 (19)
Yb1	0.04699 (10)	0.02374 (8)	0.03358 (9)	0.00377 (6)	0.00364 (6)	0.01108 (5)

### Geometric parameters (Å, °)

**Table:**

O7A—H7A	0.818 (12)	C8—C9	1.387 (5)
O7A—H7B	0.689 (6)	C8—C18	1.496 (4)
O7B—H7C	0.775 (19)	C9—C10	1.397 (4)
O7B—H7D	0.754 (17)	C9—H9	0.94
F1A—C21A	1.299 (10)	C10—N2	1.344 (4)
F2A—C21A	1.313 (9)	C10—C11	1.485 (5)
F3A—C21A	1.325 (7)	C11—N3	1.359 (4)
C21A—C22	1.573 (9)	C11—C12	1.376 (5)
F4A—C25A	1.307 (8)	C12—C13	1.391 (5)
F5A—C25A	1.348 (8)	C12—H12	0.94
F6A—C25A	1.293 (9)	C13—C14	1.381 (6)
C25A—C24	1.589 (10)	C13—H13	0.94
F7A—C26A	1.327 (7)	C14—C15	1.364 (6)
F8A—C26A	1.317 (9)	C14—H14	0.94
F9A—C26A	1.309 (10)	C15—N3	1.349 (5)
C26A—C27	1.540 (8)	C15—H15	0.94
F10A—C30A	1.323 (7)	C16—N4	1.327 (6)
F11A—C30A	1.323 (7)	C16—C17	1.386 (5)
F12A—C30A	1.313 (8)	C16—H16	0.94
C30A—C29	1.518 (8)	C17—C18	1.388 (6)
F1B—C21B	1.30 (2)	C17—H17	0.94
F2B—C21B	1.31 (2)	C18—C19	1.385 (6)
F3B—C21B	1.324 (19)	C19—C20	1.385 (5)
C21B—C22	1.41 (3)	C19—H19	0.94
F4B—C25B	1.306 (17)	C20—N4	1.331 (6)
F5B—C25B	1.352 (19)	C20—H20	0.94
F6B—C25B	1.296 (19)	C22—O1	1.242 (5)
C25B—C24	1.42 (3)	C22—C23	1.385 (6)
F7B—C26B	1.33 (2)	C23—C24	1.376 (6)
F8B—C26B	1.30 (2)	C23—H23	0.94
F9B—C26B	1.31 (2)	C24—O2	1.238 (5)
C26B—C27	1.40 (5)	C27—O4	1.266 (4)
F10B—C30B	1.32 (2)	C27—C28	1.371 (6)
F11B—C30B	1.31 (2)	C28—C29	1.386 (7)
F12B—C30B	1.30 (2)	C28—H28	0.94
C30B—C29	1.61 (4)	C29—O3	1.246 (4)
C1—N1	1.347 (5)	C31—O6	1.380 (6)
C1—C2	1.387 (6)	C31—H31A	0.97
C1—H1	0.94	C31—H31B	0.97
C2—C3	1.363 (6)	C31—H31C	0.97
C2—H2	0.94	N1—Yb1	2.438 (3)
C3—C4	1.385 (6)	N2—Yb1	2.434 (3)
C3—H3	0.94	N3—Yb1	2.464 (3)
C4—C5	1.378 (5)	O1—Yb1	2.313 (2)
C4—H4	0.94	O2—Yb1	2.319 (3)
C5—N1	1.353 (4)	O3—Yb1	2.334 (3)
C5—C6	1.478 (5)	O4—Yb1	2.262 (3)
C6—N2	1.343 (4)	O5—Yb1	2.252 (3)
C6—C7	1.390 (4)	O5—H5A	0.78 (4)
C7—C8	1.391 (5)	O5—H5B	0.75 (4)
C7—H7	0.94	O6—H6	0.83
			
H7A—O7A—H7B	123.(2)	C14—C15—H15	118.0
H7C—O7B—H7D	142.0 (16)	N4—C16—C17	123.5 (4)
F1A—C21A—F2A	107.6 (7)	N4—C16—H16	118.3
F1A—C21A—F3A	109.7 (8)	C17—C16—H16	118.3
F2A—C21A—F3A	107.6 (8)	C16—C17—C18	119.1 (4)
F1A—C21A—C22	108.9 (7)	C16—C17—H17	120.5
F2A—C21A—C22	111.1 (6)	C18—C17—H17	120.5
F3A—C21A—C22	111.9 (6)	C19—C18—C17	117.5 (3)
F6A—C25A—F4A	108.7 (8)	C19—C18—C8	121.7 (4)
F6A—C25A—F5A	107.9 (6)	C17—C18—C8	120.9 (4)
F4A—C25A—F5A	107.8 (7)	C18—C19—C20	119.4 (4)
F6A—C25A—C24	109.1 (7)	C18—C19—H19	120.3
F4A—C25A—C24	112.3 (6)	C20—C19—H19	120.3
F5A—C25A—C24	111.0 (7)	N4—C20—C19	123.2 (5)
F9A—C26A—F8A	107.3 (7)	N4—C20—H20	118.4
F9A—C26A—F7A	108.0 (7)	C19—C20—H20	118.4
F8A—C26A—F7A	107.1 (7)	O1—C22—C23	127.1 (4)
F9A—C26A—C27	110.8 (7)	O1—C22—C21B	117.2 (12)
F8A—C26A—C27	110.5 (6)	C23—C22—C21B	114.8 (12)
F7A—C26A—C27	113.0 (6)	O1—C22—C21A	113.4 (4)
F12A—C30A—F11A	105.7 (5)	C23—C22—C21A	119.5 (4)
F12A—C30A—F10A	109.7 (7)	C24—C23—C22	120.3 (4)
F11A—C30A—F10A	105.7 (6)	C24—C23—H23	119.9
F12A—C30A—C29	109.3 (5)	C22—C23—H23	119.9
F11A—C30A—C29	113.3 (6)	O2—C24—C23	127.1 (4)
F10A—C30A—C29	112.8 (5)	O2—C24—C25B	107.4 (11)
F1B—C21B—F2B	107.(3)	C23—C24—C25B	125.1 (11)
F1B—C21B—F3B	103.(2)	O2—C24—C25A	115.9 (4)
F2B—C21B—F3B	103.(3)	C23—C24—C25A	117.0 (4)
F1B—C21B—C22	120.(2)	O4—C27—C28	127.4 (4)
F2B—C21B—C22	112.(2)	O4—C27—C26B	127.(2)
F3B—C21B—C22	111.(3)	C28—C27—C26B	106.(2)
F6B—C25B—F4B	113.(3)	O4—C27—C26A	112.4 (4)
F6B—C25B—F5B	101.(2)	C28—C27—C26A	120.2 (4)
F4B—C25B—F5B	107.(2)	C27—C28—C29	122.1 (4)
F6B—C25B—C24	117.(2)	C27—C28—H28	118.9
F4B—C25B—C24	108.(2)	C29—C28—H28	118.9
F5B—C25B—C24	111.(2)	O3—C29—C28	127.0 (4)
F8B—C26B—F9B	105.(3)	O3—C29—C30A	115.7 (5)
F8B—C26B—F7B	108.(3)	C28—C29—C30A	117.2 (4)
F9B—C26B—F7B	105.(3)	O3—C29—C30B	111.8 (11)
F8B—C26B—C27	104.(4)	C28—C29—C30B	119.7 (11)
F9B—C26B—C27	120.(4)	O6—C31—H31A	109.5
F7B—C26B—C27	114.(4)	O6—C31—H31B	109.5
F12B—C30B—F11B	113.(3)	H31A—C31—H31B	109.5
F12B—C30B—F10B	111.(3)	O6—C31—H31C	109.5
F11B—C30B—F10B	100.(3)	H31A—C31—H31C	109.5
F12B—C30B—C29	118.(3)	H31B—C31—H31C	109.5
F11B—C30B—C29	111.(3)	C1—N1—C5	117.3 (3)
F10B—C30B—C29	102.(3)	C1—N1—Yb1	122.6 (3)
N1—C1—C2	123.4 (4)	C5—N1—Yb1	120.0 (2)
N1—C1—H1	118.3	C6—N2—C10	118.8 (3)
C2—C1—H1	118.3	C6—N2—Yb1	119.5 (2)
C3—C2—C1	118.4 (4)	C10—N2—Yb1	120.0 (2)
C3—C2—H2	120.8	C15—N3—C11	116.8 (3)
C1—C2—H2	120.8	C15—N3—Yb1	123.7 (2)
C2—C3—C4	119.5 (4)	C11—N3—Yb1	119.5 (2)
C2—C3—H3	120.3	C16—N4—C20	117.4 (3)
C4—C3—H3	120.3	C22—O1—Yb1	136.0 (3)
C5—C4—C3	119.3 (4)	C24—O2—Yb1	136.7 (3)
C5—C4—H4	120.3	C29—O3—Yb1	131.4 (3)
C3—C4—H4	120.3	C27—O4—Yb1	133.3 (3)
N1—C5—C4	122.1 (3)	Yb1—O5—H5A	125.(4)
N1—C5—C6	115.7 (3)	Yb1—O5—H5B	120.(4)
C4—C5—C6	122.2 (3)	H5A—O5—H5B	113.(5)
N2—C6—C7	122.0 (3)	C31—O6—H6	109.5
N2—C6—C5	116.3 (3)	O5—Yb1—O4	101.21 (10)
C7—C6—C5	121.7 (3)	O5—Yb1—O1	145.11 (10)
C6—C7—C8	119.9 (3)	O4—Yb1—O1	92.58 (10)
C6—C7—H7	120.1	O5—Yb1—O2	142.31 (11)
C8—C7—H7	120.1	O4—Yb1—O2	78.53 (10)
C9—C8—C7	117.6 (3)	O1—Yb1—O2	71.66 (9)
C9—C8—C18	121.3 (3)	O5—Yb1—O3	73.80 (10)
C7—C8—C18	121.1 (3)	O4—Yb1—O3	74.24 (10)
C8—C9—C10	119.9 (3)	O1—Yb1—O3	141.08 (9)
C8—C9—H9	120.1	O2—Yb1—O3	69.89 (9)
C10—C9—H9	120.1	O5—Yb1—N2	79.05 (9)
N2—C10—C9	121.8 (3)	O4—Yb1—N2	143.97 (10)
N2—C10—C11	116.3 (3)	O1—Yb1—N2	71.28 (9)
C9—C10—C11	121.9 (3)	O2—Yb1—N2	122.99 (9)
N3—C11—C12	122.1 (3)	O3—Yb1—N2	137.52 (10)
N3—C11—C10	115.7 (3)	O5—Yb1—N1	87.59 (11)
C12—C11—C10	122.2 (3)	O4—Yb1—N1	149.10 (10)
C11—C12—C13	119.9 (3)	O1—Yb1—N1	96.84 (10)
C11—C12—H12	120.1	O2—Yb1—N1	76.68 (10)
C13—C12—H12	120.1	O3—Yb1—N1	80.08 (10)
C14—C13—C12	118.0 (4)	N2—Yb1—N1	66.59 (10)
C14—C13—H13	121.0	O5—Yb1—N3	76.28 (11)
C12—C13—H13	121.0	O4—Yb1—N3	78.64 (10)
C15—C14—C13	119.2 (4)	O1—Yb1—N3	75.27 (9)
C15—C14—H14	120.4	O2—Yb1—N3	138.51 (10)
C13—C14—H14	120.4	O3—Yb1—N3	134.26 (9)
N3—C15—C14	124.0 (4)	N2—Yb1—N3	66.31 (9)
N3—C15—H15	118.0	N1—Yb1—N3	132.23 (9)

### Hydrogen-bond geometry (Å, °)

**Table:**

*D*—H···*A*	*D*—H	H···*A*	*D*···*A*	*D*—H···*A*
O5—H5B···Cl1^i^	0.75 (4)	2.31 (4)	3.054 (3)	175.(5)
O6—H6···Cl1	0.83	2.27	3.102 (3)	177.
O5—H5A···N4^ii^	0.78 (4)	1.92 (4)	2.686 (4)	167.(5)

Symmetry codes: (i) *x*+1, *y*, *z*; (ii) *x*, *y*+1, *z*.
